# Relationship between Fibroblast Growth Factor-23 and Mineral Metabolism in Chronic Kidney Disease

**DOI:** 10.4061/2010/167984

**Published:** 2010-12-30

**Authors:** Kosaku Nitta

**Affiliations:** Department of Medicine, Kidney Center, Tokyo Women's Medical University, 8-1 Kawada-cho, Shinjuku-ku, Tokyo 162-8666, Japan

## Abstract

Fibroblast growth factor- (FGF-)23 is a recently discovered regulator of calcium-phosphate metabolism. FGF-23 appears to decrease in synthesis and accelerated degradation of 1,25(OH)_2_D. Together with its cofactor Klotho, FGF-23 maintains serum phosphate levels within the normal range by increasing renal phosphate excretion. In chronic kidney disease (CKD), FGF-23 levels rise in parallel with the decline in renal function long before a significant increase in serum phosphate concentration occurs. Both Klotho and FGF-23, linked by a receptor mechanism, affect vitamin D synthesis and parathyroid hormone (PTH) secretion. Previous studies have shown a close association between reduced FGF-23 or Klotho activities and vascular calcification. The possible association of FGF-23 and left ventricular hypertrophy or vascular dysfunction has been proposed. Finally, prospective studies have shown that high serum FGF-23 concentrations predict more rapid disease progression in CKD patients who were not on dialysis and increased mortality in patients on maintenance hemodialysis. FGF-23 may therefore prove to be an important therapeutic target for the management of CKD.

## 1. Introduction

Fibroblast growth factor- (FGF-) 23 is recently discovered and is involved in the control of phosphate (P) and calcium (Ca) metabolism [1]. FGF-23 is a 251-amino acid protein (molecular weight; 26 kDa) that is synthesized and secreted by bone cells, mainly osteoblasts [2]. It is composed of an amino-terminal signal peptide (residues 1–24), an “FGF-like sequence” (residues 25–180), and a carboxyl-terminal extended sequence (residues 181–251), which is unique among members of the FGF family [3]. Since FGF-23 has low affinity for heparin, it can be distributed throughout the body in the blood and mediates its systemic function [4]. 

The biological activity and physiological role of FGF-23 in P and vitamin D metabolism in vivo have recently been clarified. Several animal models with excess FGF-23 activity as a result of *in vivo* forced overexpression exhibit hypophosphatemia and increased P excretion of 1,25-dihydroxyvitamin D [1,25(OH)_2_D] [5–8], and Fgf23 knockout (KO) mice are characterized by increased renal P reabsorption and an elevated serum 1,25(OH)_2_D concentration [9, 10]. FGF-23 appears to impair the synthesis and accelerate the degradation of 1,25(OH)_2_D, because expression of renal 25-hydroxyvitamin D-1*α*-hydroxylase mRNA changes within 1 h after injecting mice with recombinant FGF-23 [11]. The increased degradation of 1,25(OH)_2_D by 24-hydroxylase may be associated. Recombinant FGF-23 also has a phosphaturic effect, which is attributable to reduced renal P reabsorption. FGF-23 down-regulates the expression of both type IIa and type IIc sodium-P cotransporters on the apical surface of renal proximal tubular epithelial cells in vivo [11, 12].

## 2. Measurement of Serum FGF-23 Levels

The half-life of intact FGF-23 in the circulation of healthy individuals has been estimated to be 58 min [13]. Two assays for measurement of human FGF-23 are commercially available. One is a sandwich enzyme-linked immunosorbent assay for measurement of full-length FGF-23 that uses different monoclonal antibodies to detect the simultaneous presence of both the N-terminal [14] and C-terminal [15] portions of FGF-23. The other assay is a C-terminal assay that recognizes both full-length and processed (presumably inactive) C-terminal fragments of FGF-23. The intra-assay variability of the C-terminal FGF-23 assay is 5% at 52.7 RU/ml and 7.2% at 140.0 RU/ml, whereas interassay variability is 5% at 50.9 RU/ml and 7.3% at 153.0 RU/ml. The lower limit of detection is 3.0 RU/ml. The intra-assay variability of the intact FGF-23 assay is 4.4% at 14.6 pg/ml and 2.6% at 148.0 pg/ml, and its interassay variability is 6.1% at 15.6 pg/ml and 6.5% at 166.0 pg/ml. The lower limit of detection is 1.0 pg/ml (according to the manufacturer's specifications).

## 3. Interaction between FGF-23 and Klotho

Klotho is a 130-kDa transmembrane *β*-glucuronidase that catalyzes the hydrolysis of steroid *β*-glucuronides and was discovered by Kuro-o et al. in 1997 [16]. The Klotho gene is expressed in a limited number of tissues, mainly the kidneys, and mutations cause multiple aging-related disorders in nearly all organs and tissues [17]. Because FGF-23-KO mice exhibit phenotypes similar to those of Klotho-KO mice [18, 19], a common signaling pathway has been postulated. Indeed, FGF-23 exerts its biological effects through activation of FGF receptors (FGF-Rs) in a Klotho-dependent manner, because a Klotho/FGF-R complex binds to FGF-23 with higher affinity than FGF-R or Klotho alone [20]. FGF-23 has rather low affinity for its widely represented receptors and the presence of circulating Klotho is essential to facilitate the binding of FGF-23 to its receptors [21]. Thus, activation of FGF-23 receptors requires not only presence of the circulating FGF-23 as their ligand, but the presence of Klotho as a specific promoter whose affinity dictates the selectivity on its targets. 

Klotho is mainly expressed in the kidneys, whereas FGF-23 comes from bone cells, and this functional bone-kidney axis is of physiological and pathological relevance. Based on available knowledge, this axis seems to exert a prevailing regulation of Ca balance with Klotho and to exert a more specific and direct effect on P homeostasis through FGF-23. Both Klotho and FGF-23, linked by the receptor mechanism described above, affect vitamin D synthesis and parathyroid hormone (PTH) secretion, and both are expressed in the parathyroid glands, suggesting that FGF-23 might regulate PTH secretion. In support of this possibility, data obtained *in vitro* suggest that FGF-23 decreases PTH mRNA transcription and protein secretion in a dose-dependent manner [22]. Conversely, PTH may stimulate FGF-23 secretion by osteoblasts, because the FGF-23 levels of rodents with primary hyperparathyroidism (HPT) are increased, which may be reduced by parathyroidectomy [23].

## 4. Role of FGF-23 in Chronic Kidney Disease (CKD)

Patients with stages 3–5 CKD and dialysis patients often develop hyperphosphatemia due to impaired renal P excretion. Decreased 1,25(OH)_2_D production interferes with this process. The decrease in vitamin D activation induces hypocalcemia and a subsequent increase in PTH secretion to maintain serum Ca levels normal but induces a high level of bone turnover and hyperphosphatemia [25]. 

**Figure 1:**
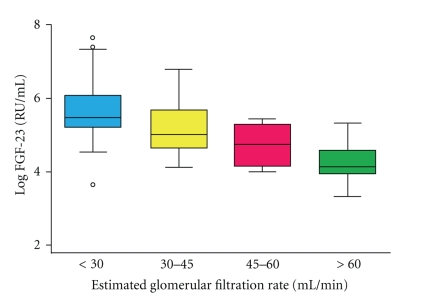
Serum fibroblast growth factor (FGF)-23 levels are negatively associated with estimated glomerular filtration rates. Cited from the J Am Soc Nephrol 2005; 16: 2205–15 by Gutiérrez et al. [24].

It has been reported that serum FGF-23 levels increased as renal function declines [26, 27]; circulating FGF-23 levels of CKD patients gradually increase with declining renal function (Figure 1) [24]. It has been therefore hypothesized that the increased FGF-23 levels in CKD are primarily the result of decreased renal clearance [28]. By contrast, no association between FGF-23 levels and glomerular filtration rate has been found in the earlier stages of CKD, when patients are normophosphatemic [28]. The hyperphosphatemia associated with CKD most likely triggers FGF-23 production, which promotes renal phosphate excretion, reflected by the severely elevated FGF-23 levels in CKD subjects. In studies of healthy subjects exposed to an increased or decreased P load, conflicting results regarding a correlation between FGF23 and serum P levels have been shown.

The cause of the increased FGF-23 levels in CKD patients is still under investigation. Instead of decreased renal clearance, there may be end-organ resistance to the phosphaturic action of FGF-23 because of a deficiency of the required Klotho cofactor. Koh et al. reported significantly reduced Klotho mRNA expression in kidney biopsy specimens of CKD patients [29]. The higher FGF-23 levels in CKD patients may reflect a physiological compensation that stabilizes serum P levels as the number of intact nephrons declines. FGF-23 increases urinary P excretion and decreases gastrointestinal P absorption both directly and indirectly by inhibiting 1*α*-hydroxylase and reducing circulating 1,25(OH)_2_D levels [30].

 Since FGF23 has a profound effect on down-regulating renal expression of the 1*α*-hydroxylase, excess FGF23 *in vivo *may signal via parathyroid cells an increase in PTH production, as a part of a counter-regulatory feedback loop to protect the decrease in vitamin D and Ca levels. There are *in vivo *animal models of FGF-23 overexpression, and all of them are characterized by parathyroid hyperplasia and increased PTH levels [32]. A more likely scenario is that the increase in PTH levels and parathyroid hyperplasia observed in these animals is mediated by the Ca-sensing receptor, again protecting systemic Ca levels, because the suppressed 1,25(OH)_2_D levels would lead to persistent hypocalcemia. Indeed, mice expressing high systemic levels of FGF-23 (R176Q) exhibit hypocalcemia and subsequent development of secondary HPT, even though their elevated PTH levels are likely to aggravate the prevailing hypophosphatemia [33].

## 5. FGF-23 and Vascular Calcification

CKD patients have dramatically higher cardiovascular morbidity and mortality rates than the general population [34]. In the last 10 years, several studies have pointed to vascular calcification as a major cause of cardiovascular disease in the dialysis population [35–39]. CKD patients develop extensive medial calcification, which causes increased arterial stiffness and high morbidity and mortality due to cardiovascular events [40]. Vascular calcification alters the pulsatile dynamics and thereby contributes to an increase in left ventricular load, and it is the most important contributor to the development of left ventricular hypertrophy in patients undergoing hemodialysis [41]. A variety of risk factors are associated with vascular calcification in dialysis patients (dialysis vintage, uremic toxins, history of diabetes, inflammation), but abnormalities in bone mineral metabolism may play a critical role [42]. In fact, elevated serum P and PTH levels contribute to vascular calcification, although their roles are incompletely understood. Elevated serum P levels may worsen cardiovascular events in the uremic population by progressively increasing calcium deposition in the coronary arteries and heart valves [43].

**Figure 2:**
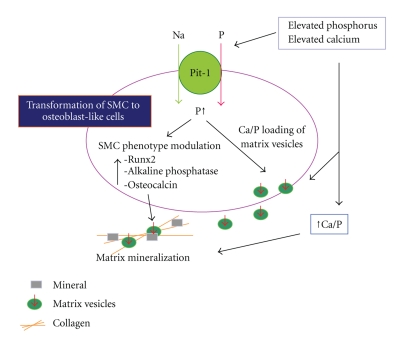
Proposed model for the effects of elevated Ca and P on vascular smooth muscle cell (SMC) matrix mineralization. Cited from the J Am Soc Nephrol 2004; 15: 2959–164 by Giachelli [31].

Jono et al. showed that elevated P levels (>2.4 mM) induce smooth muscle cell (SMC) calcification in vitro [44]. P transport into cells from the extracellular compartment is primarily mediated by membrane transporters called sodium-dependent P cotransporters, and a type III sodium-dependent P cotransporter, Pit-1, has been found to be associated with SMC calcification [45]. Likewise, increasing the Ca levels in culture media to levels that are considered hypercalcemic (>2.6 mM) leads to increased mineralization and phenotypic transition of SMCs [46]. Elevated Ca-induced mineralization is also dependent on Pit-1.  Figure 2 depicts the mechanism of vascular calcification as a result of transformation of SMC to osteoblast-like cells [31]. 

Both human and animal studies have shown a close association between reduced FGF-23 or Klotho activities and vascular calcification. Extensive vascular calcification is observed in Fgf23-KO mice by 6 weeks of age [47], interestingly, expression of the sodium-P cotransporter NaPi2a by their proximal tubular epithelial cells is upregulated. Moreover, the bone mineral density (BMD) of Fgf23-KO mice is strikingly reduced. Human studies have also shown an association between reduced BMD and vascular calcification [48], and low BMD has been suggested to independently predict coronary artery in women, with a higher odds ratio than traditional risk factors [49]. In view of the phenotypes of both Fgf23- and Kloth-KO mice, it seems likely that *in vivo* dysregulation of the FGF-23-Klotho axis can lead to vascular calcification, possibly by affecting mineral ion metabolism [50].

## 6. FGF-23 As a Prognostic Factor in CKD Patients

**Figure 3:**
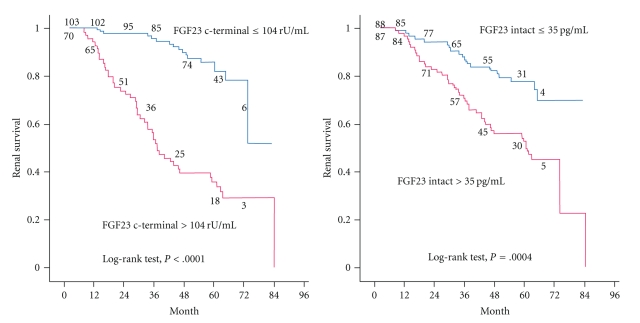
Association between serum FGF-23 levels and renal survival. Cited from the J Am Soc Nephrol 2007; 18: 2600–8 by Fliser et al. [27].

Interest in FGF-23 increased tremendously after the publication of two prospective cohort studies in which FGF-23 was identified as a risk factor for a poorer outcome in CKD patients. The ArMORR study revealed that high FGF-23 levels in patients starting hemodialysis were an independent predictor of 1-year mortality after adjustment for serum P levels [51], and in a multivariable adjusted model, subjects whose FGF-23 levels were in the highest quartile had nearly a sixfold increase in risk in mortality over the subjects in the lowest quartile. Thus, FGF-23 was a much stronger predictor of mortality than serum P levels. In the MMKD trial [27], 227 patients with nondiabetic stages 1–4 CKD were prospectively followed up for a median of 53 months to assess the progression of renal disease. Among the various parameters of Ca-P metabolism evaluated, FGF-23 levels were a significant independent predictor of CKD progression (Figure 3), which was defined as a doubling of the serum creatinine level and/or terminal renal failure. Again, the FGF-23 levels were a better outcome predictor than the serum P levels, and in a Cox regression analysis the P levels lost their predictive value after adjusting for FGF-23.

Serum FGF-23 levels have been reported to be independently related to aortic calcification index in hemodialysis patients [52]. Jean et al. reported an association between serum FGF-23 levels and increased mortality in long hemodialysis patients [53]. Multivariate linear regressions have shown a correlation between phosphatemia and serum FGF-23 levels, confirming the association between higher serum FGF-23 levels with mortality and vascular calcification regardless of the serum P levels. On the other hand, Inaba et al. found that plasma FGF-23 level of both male nondiabetic and diabetic hemodialysis patients is an independent factor negatively associated with peripheral vascular calcification in the hand artery, but not in aorta, even when adjusted for the Ca x P product [54]. Thus, serum FGF-23 levels may provide a reliable marker for Moenckeberg's medial calcification in male hemodialysis patients, independent of the regulatory effect of FGF-23 on P metabolism, followed by increased mortality in these patients [55].

## 7. Conclusion and Future Prospects

FGF-23 is a recently discovered regulator of Ca and P metabolism. Serum FGF-23 levels have been reported to increase in the very early stages of CKD, and the increase may represent a long-term, delayed response to transient episodes of hyperphosphatemia occurring in the early stage of CKD, possibly when the rapid response of PTH is becoming insufficient. Accordingly, both FGF-23 and PTH are involved in the adaptive response to ensuing secondary hyperparathyroidism, with the difference that PTH accentuates the calcitriol deficiency but FGF-23 does not.

In clinical trials, elevated FGF-23 levels have been found to be independently associated with more rapid progression of CKD, therapy-resistant secondary HPT [56], and increased cardiovascular mortality in dialysis patients. However, FGF-23 is not just an innocent bystander that reflects unfavorable derangements of Ca-P metabolism (e.g., increased serum phosphate levels) in CKD, but a pathophysiologically relevant factor. Thus, FGF-23 is a promising candidate for a therapeutic target that might improve the fatal prognosis of CKD patients. Since any increase in serum P level seems to link between CKD and cardiovascular disease, clinical interest in FGF-23 seems to be appropriate. 

It has recently been reported that FGF-23 is independently associated with left ventricular hypertrophy in patients with CKD [57]. In addition, circulating FGF-23 has been associated with vascular dysfunction in the community [58]. Additional studies are required to determine possible direct effects on the left ventricle and vasculature. A recent study that evaluated serum FGF-23 levels in a cohort of patients starting dialysis found that its quartiles are progressively and independently associated with mortality. Moreover, FGF-23 may be a cause of 1,25(OH)_2_D insufficiency, and that could be a further link with increased mortality, in view of the role of vitamin D receptor activation that is claimed to exist in several disease conditions. Finally, the functional interplay between FGF-23 and Klotho may represent another link with mortality.
